# Identification of Widespread Ultra-Edited Human RNAs

**DOI:** 10.1371/journal.pgen.1002317

**Published:** 2011-10-20

**Authors:** Shai Carmi, Itamar Borukhov, Erez Y. Levanon

**Affiliations:** 1 The Mina and Everard Goodman Faculty of Life Sciences, Bar-Ilan University, Ramat-Gan, Israel; 2 Compugen Ltd., Tel-Aviv, Israel; University of Washington, United States of America

## Abstract

Adenosine-to-inosine modification of RNA molecules (A-to-I RNA editing) is an important mechanism that increases transciptome diversity. It occurs when a genomically encoded adenosine (A) is converted to an inosine (I) by ADAR proteins. Sequencing reactions read inosine as guanosine (G); therefore, current methods to detect A-to-I editing sites align RNA sequences to their corresponding DNA regions and identify A-to-G mismatches. However, such methods perform poorly on RNAs that underwent extensive editing (“ultra”-editing), as the large number of mismatches obscures the genomic origin of these RNAs. Therefore, only a few anecdotal ultra-edited RNAs have been discovered so far. Here we introduce and apply a novel computational method to identify ultra-edited RNAs. We detected 760 ESTs containing 15,646 editing sites (more than 20 sites per EST, on average), of which 13,668 are novel. Ultra-edited RNAs exhibit the known sequence motif of ADARs and tend to localize in sense strand Alu elements. Compared to sites of mild editing, ultra-editing occurs primarily in Alu-rich regions, where potential base pairing with neighboring, inverted Alus creates particularly long double-stranded RNA structures. Ultra-editing sites are underrepresented in old Alu subfamilies, tend to be non-conserved, and avoid exons, suggesting that ultra-editing is usually deleterious. A possible biological function of ultra-editing could be mediated by non-canonical splicing and cleavage of the RNA near the editing sites.

## Author Summary

The traditional view of mRNA as a pure intermediate between DNA and protein has changed in the last decades since the discovery of numerous RNA processing pathways. A frequent RNA modification is A-to-I editing, or the conversion of adenosine (A) to inosine (I). Since inosine is read as a guanosine (G), A-to-I editing leads to changes in the RNA sequence that can alter the function of its encoded protein. In recent years, tens of thousands of human A-to-I editing sites were discovered by computationally comparing RNA sequences to the human genome and searching for A-to-G mismatches. However, previous screens usually ignored RNA sequences that were edited to extreme, because the large number of A-to-G mismatches carried by these RNAs obscured their genomic origin. We developed a new computational framework to detect extreme A-to-I editing, or ultra-editing, based on masking potential editing sites before the alignment to the genome. Our method detected about 14,000 editing sites, with each edited molecule affected, on average, in more than 20 nucleotides. We demonstrated that the likely reason for the ultra-editing of those sequences is their potential to fold back into a particularly long double-stranded structure, which is the preferred target of the editing enzymes.

## Introduction

Post-transcriptional modification of RNA molecules increases the complexity of the transcriptome and constitutes an additional mechanism for controlling gene activity. One of the most frequent modifications in primates is Adenosine-to-Inosine (A-to-I) RNA editing of pre-mRNA. Since inosine is later translated as guanosine (G), A-to-I editing can lead to recoding of protein sequences. A-to-I editing, mediated by adenosine deamisnase proteins acting on double-stranded RNA (ADARs) [1]–[4], is crucial for normal life and development [5], [6] and was found to play a role in human disease, especially brain related [7], [8]. Editing affects gene expression, both globally and in a gene-specific manner [9]–[14], and enhances the cell's capacity of information processing and evolvability [15], [16]. Inosine is recognized as guanosine also during sequencing; editing can therefore be detected as a G in an RNA sequence with an A in the corresponding genomic DNA. Systematic surveys of cDNA and EST libraries [17]–[25], as well as experimental genome-wide screens [26]–[29], have so far detected about 40,000 human editing sites [30].

Known A-to-I editing sites can be roughly classified into two categories. In the first type, specific sites are edited in coding sequences. This type of editing usually modifies a protein sequence and potentially its function, and is therefore highly selective: in each gene, only one or few, specific, usually conserved sites are edited, in a regulated manner. Only few tens of such editing sites are currently known [2]. In the second category, which encompasses the bulk of the sites, adenosines at repetitive elements are indiscriminately hyper-edited, mostly in Alu elements [31] in UTRs or introns [17]–[21]. Due to the large number of Alu repeats in the human genome, adjacent, reversely oriented Alus can form double stranded RNA (dsRNA) structures that serve as targets for ADAR proteins. Editing of repetitive elements is highly promiscuous and ranges between a few to tens of nucleotides. The biological role of hyper-editing is mostly elusive. However, a few functions were proposed. For example, a hyper-edited RNA was shown to be retained in the nucleus [10] and to be released upon cleavage [14]. Inosine-containing synthetic dsRNAs were shown to be cleaved at specific sequences [32], to globally down-regulate gene expression [13], and to suppress apoptosis [33]. Changes in the RNA sequence, even if outside coding sequences, can also be functional, if, for example, they occur at splice sites [34], [35] or at miRNA targets [36].

A particularly interesting class of hyper-edited RNAs, which we refer to here as ‘ultra’-edited RNAs, represents molecules that underwent editing of an extremely large fraction of their adenosines (for a precise definition see Materials and Methods). Although it is known that long synthetic dsRNAs are ultra-edited [37]–[39], not much is known about such endogenous RNAs— except for a small number of ultra-edited RNAs that were occasionally discovered (e.g., in [17], [19], [40]–[42]), ultra-editing was usually overlooked in systematic RNA editing detection screens. These methods work by aligning candidate RNA sequences to the reference genome and searching for clusters of A-to-G mismatches. However, for extensively edited RNAs, the alignment to the genome suffers from so many mismatches that the RNA is likely to be discarded. Based on this observation, on the preliminary evidence for ultra-edited RNAs, and on the large amount of cellular inosine [43], we suspected that many more ultra-edited RNAs exist.

In this paper, we devised and applied a computational pipeline to identify ultra-edited RNA. We started with RNA sequences that previously could not be aligned to the genome, and realigned them after reducing the genomic DNA and RNA sequences to three letters by an A→G transformation. This way, mismatches in ultra-edited RNAs due to A-to-I editing were masked and fast alignment algorithms could be employed to detect the genomic origins of these RNAs. Whenever a transformed RNA has successfully aligned to the transformed genome, the original sequences were recovered and the mismatches were examined. A particularly large number and density of A-to-G mismatches indicated that the RNA was ultra-edited. We detected, with high confidence, 760 ultra-edited RNAs edited in over 14,000 editing sites, most of which were previously unknown. Comparison of the ultra-edited elements with sites of moderate editing suggested that, as expected, ultra-editing is preferred in repeat-rich regions with potential for particularly long fold-back dsRNA structure.

## Materials and Methods

**Figure 1:**
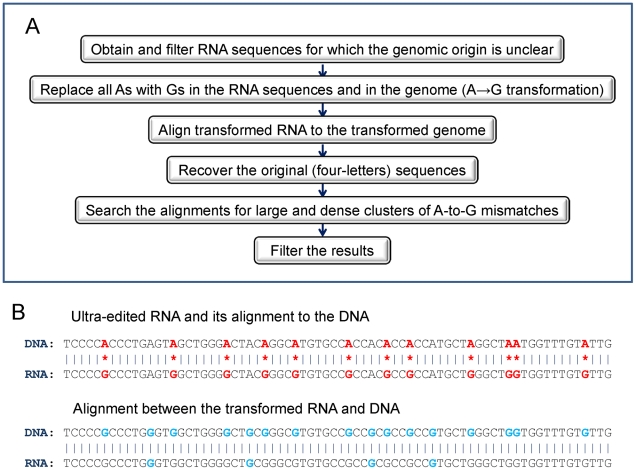
The computational procedure used to detect ultra-edited RNA. (A) An outline of the procedure. (B) An illustration of the transformation algorithm. Top panel: an alignment between (hypothetical) ultra-edited RNA and its DNA source. A-to-G mismatches are denoted with red stars and mismatching nucleotides are highlighted in red. Bottom panel: alignment of the same sequences, but where each A was transformed to G (in both the DNA and the RNA). Transformed nucleotides are highlighted in light blue. A-to-G mismatches, but also A-A matches, become G-G matches in the transformed sequences. The transformed DNA and RNA therefore perfectly align.

The computational procedure for detecting ultra-edited RNA is described below (overviewed in Figure 1A).

### Extraction of candidate sequences

We queried the UCSC Genome Browser [44] (http://genome.ucsc.edu) for long (>250 bp) human ESTs or mRNAs from GenBank that did not align to the genome, and downloaded their sequences from NCBI Batch Entrez (http://www.ncbi.nlm.nih.gov/sites/batchentrez). The 458,124 sequences were filtered to discard possible low-quality sequences: ESTs or mRNAs with particularly large (>60%) or small (<10%) percentage of a single nucleotide, with over 10% of ambivalent nucleotides (non-[ACGT]), or with over 50% simple repeats content. We also aligned (MEGABLAST [45]; http://www.ncbi.nlm.nih.gov/blast/megablast.shtml) the remaining 438,807 sequences to the genome (GRCh37/hg19) and eliminated each sequence that aligned with ≥98% identity (along ≥90% of its length). The remaining 334,344 candidate sequences were sent to downstream analysis. Since the number of full-length mRNAs was relatively small (∼2%), we refer henceforth to our candidate sequences as ESTs, or just RNAs, interchangeably.

### DNA and RNA transformation

A-to-I ultra-edited RNAs harbor a large number of A-to-G mismatches (A in the DNA, G in the RNA), but no (or very few) mismatches of any other type. Therefore, an ultra-edited RNA would generate a good alignment to the genome (and therefore be detected) if A-to-G mismatches will be specifically ‘masked’. To this end, we transformed every A to G both in the genomic DNA sequence and in the candidate RNA sequences. As demonstrated in Figure 1B, ultra-edited, high-quality, transformed RNA sequences will align perfectly to the transformed DNA. Low-quality, erroneous RNA sequences will not align well even after the transformation.

A-to-I editing always takes place on the sense strand. However, the actual sequenced DNA and RNA strands are arbitrary. Therefore, to detect all ultra-edited RNAs, all strand combinations must be separately aligned (DNA+/RNA+, DNA+/RNA−, DNA−/RNA+, DNA−/RNA−; see Table S1). For genuine ultra-edited RNA, exactly one strand combination will produce a good alignment after the transformation. Note that additional information on transcription direction (e.g., a polyA tail, protein sequence, splicing signals, etc.) is required to rule out the possibility that the A-to-G mismatches are due to a T-to-C editing event (see also Table S1).

With A→G transformation, we detect clusters of A-to-G mismatches, but also clusters of G-to-A. The G-to-A clusters serve as a negative control, because we expect such clusters to result from a sequencing error. The same holds true for other types of mismatches; we therefore created additional transformations: A→C (×4 strand combinations), G→C (×2), and A→T (×2). For G→C and A→T, it is sufficient to align the (+) DNA to the (+/−) RNA, as the other two combinations (with (−) DNA) are equivalent to the first two. The 12 transformations are summarized in Table S1.

### Alignment of the transformed sequences

To speed up the computation of the alignments, we uploaded the candidate RNA sequences and the human genome to a commercial cloud computer (http://aws.amazon.com/ec2). We performed the transformations listed above and aligned, in parallel, the 12 transformed RNA and DNA pairs using MEGABLAST [45]. We retained only the best alignment, and only when it was particularly convincing (E-value≤10^−50^, percent identity≥95%, length≥100 bp). The number of successful alignments was 690,495, ∼17% of the number of possible alignments (334,344 candidate sequences ×12 transformation/strand combinations).

### Identification of ultra-editing

For each aligning RNA and DNA pair, we realigned the original, 4-letter sequences and recorded all mismatches. Consider, for example, alignments coming from the A→G transformed sequences. We designated an RNA as ultra-edited if it satisfied the following conditions:

1. The alignment had at least 12 A-to-G mismatches.
2. The number of A-to-G mismatches was more than 90% of all mismatches.
3. The number of A-to-G mismatches was at least 20% of the number of As in the (genomic) subsequence extending from the first to the last A-to-G mismatch.

A similar procedure was used to search for RNAs with other possible types of ‘editing’ (G-to-A, A-to-C, etc.). The values of the cutoffs were chosen to roughly match the expected number of mismatches in an EST aligning to an Alu element that was discarded by UCSC (4% dissimilarity×300 bp Alu length = 12 mismatches, which are ∼20% of the ∼60 adenosines in the consensus Alu (Repbase [46])). However, as there is no clear-cut boundary between ultra-edited RNAs and other edited RNAs, other values could have been selected as well.

### Filtering of the results

RNAs passing the above criteria were further filtered to remove the following cases, where apparent editing is likely an artifact.

1. RNAs that appeared ultra-edited in more than one transformation/strand combination.
2. RNAs in which the aligning part of the RNA or DNA was too homogeneous (e.g., a single nucleotide repeat was longer than 36 bp, or one nucleotide frequency was outside the “normal” range [10%–60%]).
3. RNAs in which the alignment had too many gaps (>5 overall, or >3 in the RNA or DNA).
4. RNAs in which another MEGABLAST search against the (non-transformed) genome yielded a better alignment in another locus (over length ≥90% of that of the original alignment).
5. RNAs in which A-to-G ultra-editing was found on a particular strand of the DNA, but other mRNA sequences (from the UCSC Genome Browser) supported, by at least two sequences, transcription only from the opposite strand. This step practically served to eliminate T-to-C editing.

In total, 760 RNA sequences containing 14,538 unique editing sites survived the cleanup procedure to constitute our final set of A-to-I ultra-edited RNAs. A complete list of the ultra-edited RNAs, along with some of their properties (e.g., GenBank accession, genomic coordinates, location of mismatches, sequence context, etc.), can be found in Dataset S1. A list of the ultra-editing sites formatted as a UCSC genome browser track is given in Dataset S2.

Clearly, the pool of ESTs we analyzed contains many RNAs which are hyper-edited, even if not ultra-edited according to our strict definition. Rerunning our screen exactly as above, but allowing for less than 12 editing sites (but at least five), we discovered 280 additional ESTs containing 2,286 unique editing sites. Although a detailed analysis of these ESTs is beyond the scope of this paper, we report their coordinates and basic individual and genome-wide properties in Dataset S3, Dataset S4 and Text S1.

## Results

### Computational identification of ultra-edited RNAs

**Figure 2:**
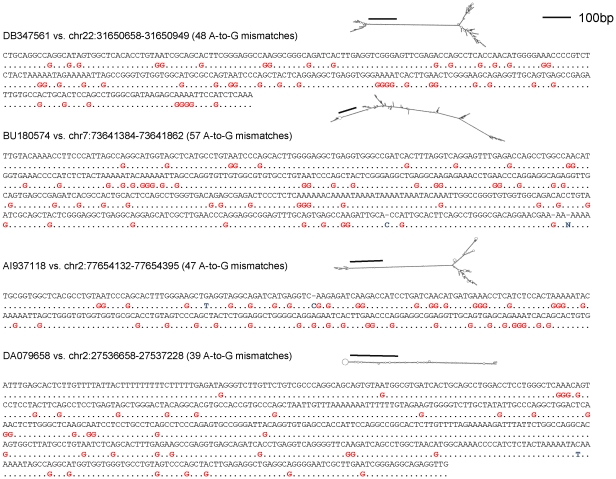
The alignment of typical ultra-edited RNAs to the genome. The alignments were generated using NCBI BLAST (http://www.ncbi.nlm.nih.gov/BLAST/Blast.cgi). The RNA secondary structure (RNAFold [48]) is also shown. The bar indicates approximately 100 base pairs. All ESTs display tens of A-to-G mismatches as well as a clear dsRNA structure.

**Figure 3:**
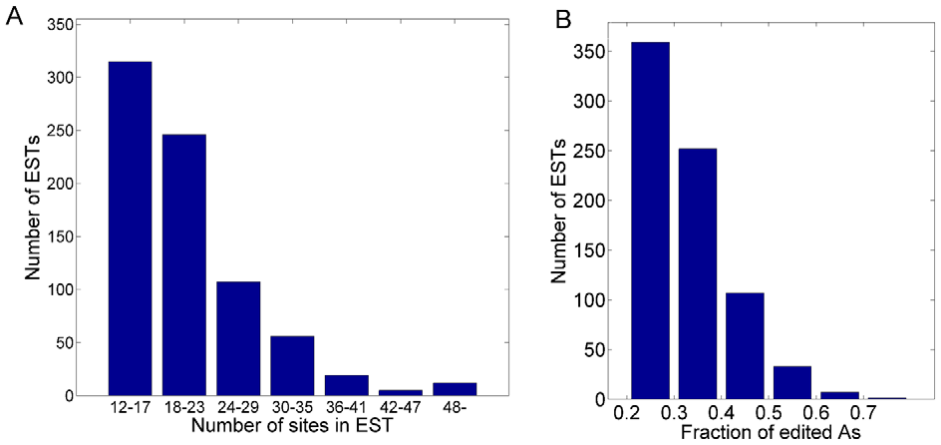
The number of editing sites and the editing rate in the ultra-edited ESTs. (A) The number of ESTs with a given number of A-to-G editing sites. (B) The number of ESTs with a given fraction of edited adenosines (“editing rate”).

We speculated that published cDNA sequences that could not be confidently aligned to the genome include some ultra-edited RNAs. We therefore extracted, from the UCSC genome browser, ∼450,000 ESTs whose genomic origin could not be confidently established, from which we removed ∼100,000 sequences with potential sequencing errors (e.g., long single-nucleotide stretches). We masked A-to-I editing sites by transforming every A to G in the RNA sequences and in the genome, and then aligned the transformed RNA and DNA sequences using MEGABLAST. We repeated the transformation and alignment for all possible strand combinations, and for other types of possible ‘editing’ (e.g., A-to-C) as a control (see Table S1). For ESTs for which a good alignment was found, the original (non-transformed) sequences were recovered and the mismatches were examined. We designated an EST as ultra-edited if the number of A-to-G mismatches was at least 12, and at least 90% of all mismatches, and if the fraction of edited adenosines to all adenosines was at least 20%. Finally, we discarded seemingly ultra-edited RNAs whose alignment was suspicious (e.g., too many gaps, high repeat content, strand ambiguous or inconsistent with other ESTs, etc.). More details on the computational procedure appear in Materials and Methods (see also Figure 1). At the termination of the computational pipeline, we remained with a final set of 760 (A-to-G) ultra-edited ESTs. Four typical cases of ultra-edited ESTs are presented in Figure 2. The distributions of the number of editing sites and the editing rates (fraction of edited As/number of As) are shown in Figure 3. Additional 280 ESTs were found when we allowed for a smaller number of editing sites in each EST. These ESTs are reported and analyzed in Dataset S3, Dataset S4 and Text S1, but are not further discussed here.

### Ultra-edited ESTs are unlikely to be a sequencing error

**Figure 4:**
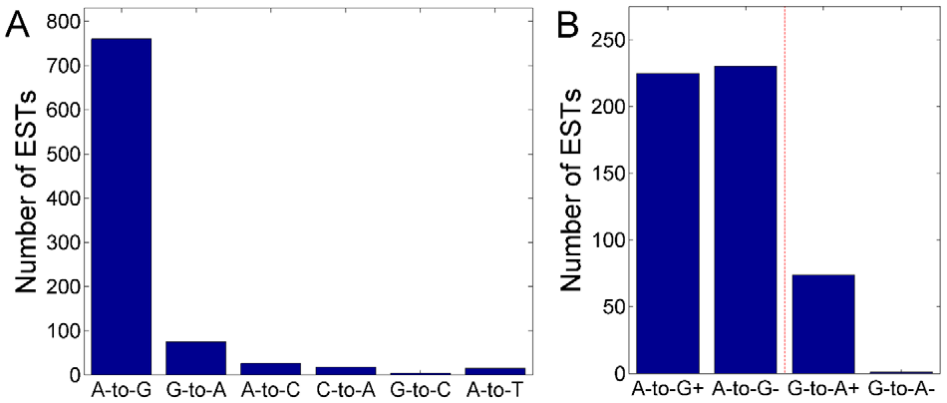
The number of ultra-editing events by mismatch type and strand. (A) The number of ultra-edited ESTs of each mismatch type. The number of A-to-G ESTs is much larger than any other mismatch type, suggesting that the A-to-G clusters are due to A-to-I ultra-editing. Only six (out of 12) mismatch types are presented: ultra-editing of the complementary mismatches were mostly removed in the cleanup procedure. (B) The number of ultra-edited ESTs of type A-to-G and G-to-A, broken by the RNA strand. The (+) sign corresponds to the sequenced RNA being A or G; the (−) sign corresponds to T or C. For G-to-A, in all but one EST the (+) strand was edited, suggesting that many G-to-A ultra-edited ESTs may be due to a sequencing error. In this panel, we excluded 305 A-to-G edited ESTs arriving from a particular library (human liver regeneration after partial hepatectomy; see the main text), since in this library almost all ESTs (edited and non-edited) aligned to the sense strand. In the NCI-CGAP libraries, from which most of the G-to-A edited ESTs came, the sequenced RNA was biased towards the antisense strand, indicating that the difference between (+) and (−) demonstrated in the plot is not due to the experimental protocol.

The number of ultra-edited ESTs of each type of mismatch is shown in Figure 4A. The number of ultra-edited ESTs of type A-to-G is more than five times the number of ‘edited’ ESTs of all other types combined (760 vs. 138). The largest class of non-A-to-G editing is G-to-A, containing 75 ESTs. To explain the origin of these 75 ESTs, we plot in Figure 4B the number of ESTs in which the edited RNA strand was (+) (the sequenced strand) or (−). Since ESTs are derived from double-stranded cDNA clones, the strand that was sequenced is usually arbitrary (relative to the sense strand), and we expect to see roughly equal numbers of (+) and (−) ESTs. However, as can be seen in Figure 4B, all but one of the G-to-A ultra-edited ESTs are from the (+) strand. This indicates that the source of these mismatches is possibly a technical sequencing error [21]. In support of this hypothesis, we note that the vast majority (63/75) of the G-to-A ESTs came from NCI-CGAP (National Cancer Institute – Cancer Genome Anatomy Project) libraries, as opposed to just 99/760 for A-to-G. Additionally, 65/75 of the ESTs were sequenced in the year 1997, compared to only 114/760 for A-to-G. It is thus conceivable that most of the G-to-A clusters are due to isolated cases of technical faults.

### Most A-to-G editing sites are novel

The total number of A-to-G editing sites discovered by our screen is 15,646, of which 14,538 are unique. This the same order of magnitude as discovered in former editing screens [17]–[19]. Almost all sites (13,668, 94%) are novel: they did not appear in DARNED [30], the most up to date database of RNA editing in humans. The 760 ultra-edited ESTs map to 695 distinct genomic regions, 647 of which are covered by one ultra-edited EST, 41 by two ESTs, and one (chr3:183879216–183879642+, intron of DVL3 gene) by 11 ESTs (all from the lung EN0096 library). Only 42 sites (0.29%) overlap with genomic SNPs.

### The ultra-editing sequence motif is similar to the known ADAR1 motif

**Figure 5:**
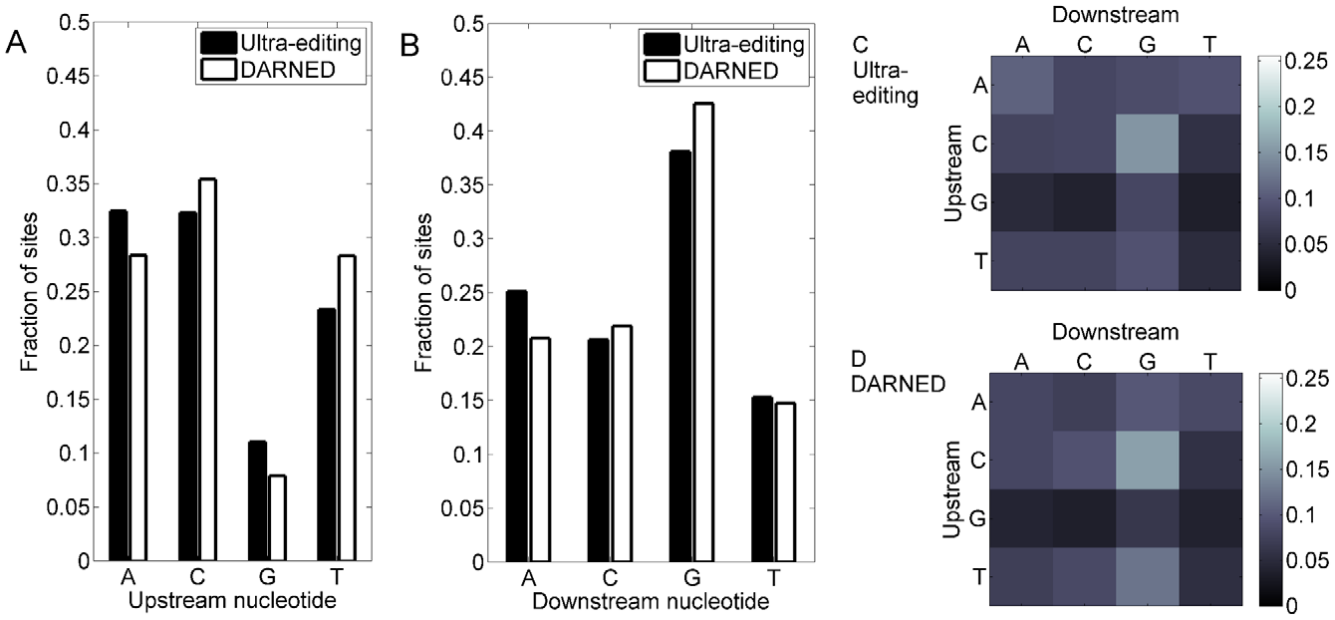
Sequence context of ultra-editing. (A) The composition of (genomic) nucleotides upstream of the editing sites. Solid bars- ultra-editing sites; hollow bars- all previously known editing sites (from DARNED, the database of RNA editing [30]). Shown is the fraction of sites with each type of nucleotide. (B) Same as (a), for the nucleotide downstream of the editing site. The main editing motif for both DARNED and ultra-editing is a deficit in G upstream and an excess of G downstream of the editing site. (C) The fraction of each dinucleotide combination (upstream-downstream) for the ultra-editing sites. Brighter squares correspond to more frequent dinucleotides (color coded on the right). (D) Same as (c) for DARNED.

Figure 5 shows the frequency of nucleotides upstream and downstream of the editing sites, as well as the frequencies of their combinations. The sequence preference of all previously known editing sites (as listed in DARNED) is also presented. As expected [17]–[20], [26], [27], guanosines are depleted upstream and overrepresented downstream of the editing sites. The frequencies of the other nucleotides differ slightly between ultra-editing and DARNED, particularly for upstream As and Ts. Comparison of all dinucleotide combinations between the ultra-editing sites and the DARNED sites reveals that ultra-editing is relatively more common than DARNED at AAA, GAA, and GAG (the middle A is the editing site) and is less common than DARNED at CAC, AAG, and TAG. The latter two are ADAR2 motifs [39], suggesting that ultra-editing is mediated mostly by ADAR1.

### Tissues enriched in ultra-editing

We next characterized the conditions under which ultra-editing has occurred. A list of the ultra-edited tissues and health states, sorted by the number of edited ESTs, is given in Table 1. The most surprising observation is the large amount of ultra-edited ESTs in the liver. Further investigation revealed that 305 of these ESTs are from a single library named “Human liver regeneration after partial hepatectomy” (Library ID:18893). We believe that these ESTs represent *bona fide* A-to-I editing events for the following reasons. First, the fraction of ESTs not aligning to the genome (http://genome.ucsc.edu/) in the liver library is neither exceptional nor even the largest. The fraction of non-aligning ESTs that are ultra-edited is also not the largest. Next, the sequence context of the liver ultra-editing sites is the one expected from ADAR targets, namely, a deficit of G upstream and an excess of G downstream of the editing site. Finally, all but seven of the liver ultra-edited ESTs overlap with an Alu element. We thus speculate that the ultra-edited liver library has been generated under experimental conditions of ADAR overexpression, perhaps due to induction by interferon [47]. Of the other tissues, brain is the most ultra-edited, followed by lung, thymus, and eye. In Table 1, we also report the enrichment factor of each tissue, that is, the number of ultra-edited ESTs in the tissue divided by the expected number. The tissues most enriched are thymus, spleen, muscle, and brain. Ultra-editing in cancer tissues is infrequent [48].

**Table 1:** Top tissues and health states containing ultra-edited ESTs.

Tissue	Number of ESTs	Enrichment^a^	Health state	Number of ESTs	Enrichment^a^
Liver	312	12.98	Normal	563	1.57
Brain	118	0.97	Lung tumor	13	0.73
Lung	33	0.89	Glioma	9	0.78
Thymus	31	3.74	Soft tissue/muscle tissue tumor	9	0.69
Eye	21	0.93	Non-neoplasia	8	0.72
Muscle	20	1.63	Head and neck tumor	8	0.37
Prostate	20	0.65	Colorectal tumor	8	0.4
Uterus	19	0.74	Kidney tumor	6	0.61
Uncharacterized tissue	15	0.4	Gastrointestinal tumor	6	0.42
Spleen	12	2.17	Uterine tumor	6	0.57

a The enrichment is the number of ultra-edited ESTs from the tissue divided by the expected number, which was computed as follows. For each tissue, we calculated the ratio of the total number of ESTs in the tissue to the total number of ESTs in all tissues. The expected number of ultra-edited ESTs in a tissue is the latter ratio multiplied by the total number of ultra-edited ESTs. Enrichment of health states was similarly calculated.

### Most ultra-edited RNAs overlap with relatively new Alu elements

As expected, almost all ultra-edited RNAs overlapped with an Alu element (693/760), and only six did not overlap with any repeat. An important question raised by our finding of ultra-edited RNAs is whether these RNAs have any distinct properties. To address this question, we compiled, using DARNED, a list of all previously known A-to-I editing clusters that are not ultra-edited, by grouping adjacent editing sites (separated by less than 300 bp, the Alu length) and eliminating clusters with a single site or with 12 or more sites. This resulted in a set of 4456 “short clusters” to which we compared our ultra-edited ESTs. In Table 2, we report the fraction of edited RNAs originating from each major Alu sub-family (AluJ, AluS, and AluY). Most notably, ultra-edited ESTs are underrepresented in AluJ elements (P<10^−14^, χ^2^-test comparing AluJ elements to all others). In comparison, the number of DARNED's short clusters found in AluJ elements is roughly what is expected based on the genome-wide distribution of these elements (P = 0.64; χ^2^-test). As AluJ is the oldest Alu sub-family, these results suggest that ultra-editing sites were eliminated from relatively old Alu sub-families.

**Table 2:** The fraction of edited elements from each major Alu sub-family.

Alu sub-family	Number of ultra-edited ESTs^a^	Number of DARNED short clusters	Total number in the entire genome
AluY	91 (11.2%)	415 (9.4%)	143,178 (12.6%)
AluS	601 (73.9%)	2811 (63.6%)	686,962 (60.1%)
AluJ	121 (14.9%)	1194 (27%)	312,138 (27.3%)

a Note that the sum of the second column exceeds the number of ultra-edited ESTs because some ESTs overlap with more than one Alu.

### Strand preference of the ultra-edited Alus

The strand of an Alu element within a transcript can be either sense or antisense. We found that ultra-edited Alu elements have a clear strand preference: 630 ultra-edited Alus are sense (77%), compared to only 186 antisense (23%). In DARNED's short clusters, there is almost no strand preference: 2382 sense (53%) vs. 2141 antisense (47%). The explanation of this result is likely the composition bias of the Alu elements: even without the terminal polyA tail, the consensus sense strand Alu (Repbase [46]) has 59 As compared to only 46 Ts.

### Ultra-editing substrates form relatively long dsRNA structure

We speculated that ultra-editing occurs at particularly long or stable dsRNA structure [37], [38], [40], [49]. We therefore calculated the maximum possible length of dsRNA structure in the edited regions. We used two measures: the total number of matching base pairs when aligning the edited region and its reverse complement, and the maximal length of the stem in the RNA secondary structure, as predicted by RNA Fold [50]. Indeed, the putative dsRNA length is significantly longer, according to both measures, in the ultra-edited regions than in DARNED's short clusters (Table 3, properties 1,2). The reason for the increased dsRNA length is likely the dramatic overabundance of repeats in the ultra-edited flanking regions (Table 3, property 3). Specifically, the ultra-edited regions have a larger number of inverted pairs of Alu repeats than the short clusters (Table 3, property 4), and a smaller distance between the edited Alu and the nearest inverted Alu (Table 3, property 5).

**Table 3:** Secondary structure and repetitive elements in the edited regions.

	Property	Ultra-editing^c^^,^ d	DARNED short clusters^c^^,^ d	P-value^e^
1	Maximum length of dsRNA using BLAST^a^.	322±11	212±4	9.6×10^−23^
2	Maximum length of dsRNA using RNA Fold^b^.	400±5	363±2	2×10^−12^
3	Total repeat content in the region.	63.2%±0.7%	52.6%±0.3%	6×10^−37^
4	Minimum of (number of +Alus, number of −Alus) in the region.	3.8±0.09	3.59±0.04	2.6×10^−2^
5	Distance between the edited Alu and the nearest inverted Alu.	855±52	956±21	3.4×10^−7^

a The edited region and its reverse complement were aligned using BLAST. We used the total number of aligning base pairs as an estimate of the length of the dsRNA.

b The secondary structure of the RNA was calculated using RNAFold [50]. We used the maximal number of open brackets in the structure as an estimate of the length of the dsRNA.

c Regions used: 1.5 kbp flanking upstream and downstream of the edited regions for properties 1,2, and 3; 5 kbp for property 4.

d Means are reported along with the standard error of the mean [sqrt(sample variance/n)].

e P-values were calculated using Mann-Whitney U test.

### Ultra-edited sites are relatively rare in exons

Most ultra-edited RNAs overlap with genes (547/760 ESTs (72%); the overlap is with 460 genes; gene annotation is from the UCSC genome browser). Among these, 61 (8%) overlap with exons: 38 with 3′UTRs, four with 5′UTRs, 17 with non-coding RNA, and two with coding sequences (DW412140 with GSK3B and DA857874 with OLR1). The other 486 ESTs overlap with introns. The higher level of editing in 3′UTRs compared to 5′UTRs, which has been previously observed [17], [20] and is also observed in the DANRED database, is most probably due to their larger sizes (mean ∼525 bp, compared to ∼145 bp for the 5′UTR [51]). DARNED's short clusters have only slightly larger overlap with genes (75% (3359/4456); P = 0.02, binomial test), but significantly larger overlap with exons (1239/4456 (28%); P = 10^−42^; binomial test). A list of the ultra-edited ESTs overlapping with exons is given in Dataset S5. A functional classification of the ultra-edited genes appears in Dataset S6. Among the ultra-edited genes, 19 are related to stress response, 14 to apoptosis, and three to hematopoiesis (also listed in Dataset S6), which could be related to the known role of ADAR1 in these processes [5], [52]–[56].

### Possible cleavage of ultra-edited RNAs

Hyper-edited RNAs can be specifically cleaved [32], [57], [58], and hundreds of putative hyper-editing sites were shown to be non-canonically (NC) spliced out of UTRs [59]. To find out if ultra-edited regions are also cleaved or NC-spliced, we searched for ultra-edited regions that overlap with both a UTR and an intron. We found 31 such ESTs, listed with their genes in Dataset S7. We manually inspected the splice variants of these genes to identify cleavage or NC-splicing. Cleaved RNAs appear as properly spliced sequences, up to a certain point where an exon extends abnormally until it is cleaved at the ultra-edited region. NC-spliced RNAs also appear to be normally spliced, except for an additional short intron in their 3′UTR, whose boundaries overlap with the ultra-edited Alus but lack the GT-AG canonical splicing signals. We identified ten cleaved and five NC-spliced mRNAs in regions of ultra-editing (indicated in Dataset S7), including one that was previously shown [59]. We note that few of the cleavage sites may be alternatively explained as premature polyadenylation at the Alu sequence [60], [61].

### Ultra-edited genomic regions are slightly less conserved than moderately edited regions

Ultra-editing substrates are more abundant in introns and in new Alu sub-families than the short clusters, indicating their general adverse effect. We hypothesized that ultra-edited genomic regions are also less conserved. Therefore, we extracted for each edited region (with flanking 500 bp upstream and downstream), the average primate PhyloP [62] conservation score, which is a measure of the acceleration or reduction of the rate of nucleotide substitution. The ultra-edited regions are less conserved (average score 8*10^−3^ (±2*10^−3^ standard error of the mean)) compared to the short clusters ((15±1)*10^−3^; P = 0.004 (t-test)). We note though that when comparing an alternative conservation score (PhastCons [63], which is the probability the entire region is conserved), no difference is observed between ultra-editing sites and short clusters.

### Experimental validation of ultra-edited RNAs

**Figure 6:**
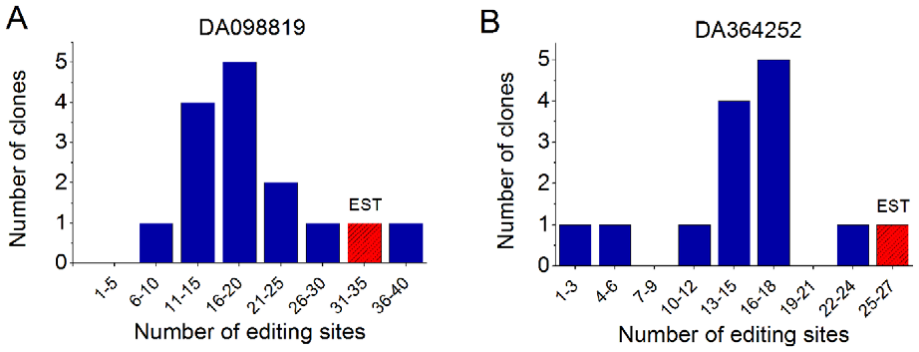
Experimental validation of an ultra-edited RNAs. We experimentally validated ultra-editing in the ESTs DA098819 (A) and DA364252 (B). We generated cDNA from cerebellum RNA and amplified cDNA fragments that correspond to chr19:53120654–53121052 (A) and chr2:242643522–242644012 (B). We cloned the PCR products, sequenced the clones (14 in (A), 13 in (B)), and aligned the sequences to the genomic DNA. In the figure, we show the number of clones with each given number of editing sites. The red, striped bar in each panel indicates the number of sites in the EST. Almost all clones are highly edited, with at least one clone edited to about the same extent as the ultra-edited EST.

We selected two ultra-edited RNAs, for which no editing was known before, for experimental validation. The first EST, DA098819, was derived from an AluSx element in the intron of the ZNF83 gene (chr19:53120521–53121009−). It was generated from a normal brain and had 34 A-to-G mismatches. The second EST, DA364252, came from an AluSq element in the intron of ING5 (chr2:242643522–242644012+). It was also generated from a normal brain and had 25 mismatches. We amplified genomic DNA and cDNA from a brain of a single donor for these two targets (details on experimental procedures are given in Text S2). The genomic DNA was sequenced, and the cDNA PCR product was cloned. We selected and sequenced several clones (14 for DA098819, 13 for DA364252) and searched for A-to-G mismatches when compared to the genomic DNA. For DA098819, the average number of A-to-G mismatches per clone was 19, with the most heavily edited clone having 36 mismatches. The total number of editing sites we found (over all clones) was 45; these sites cover 33/34 of the sites seen in the EST. For DA364252, the average number of sites was 14, with 22 sites in the most edited clone. Over all clones, 38 sites were found, covering 19/25 of the sites of the EST. A histogram of the number of clones with each number of editing sites for the two targets is presented in Figure 6. The alignment of the clones to the genomic DNA, annotation of the editing sites, and additional statistics appear in Dataset S8.

## Discussion

Previous screens to detect RNA editing systematically overlooked RNA sequences that poorly aligned to the genome. We conjectured that many of these sequences are in fact highly edited and therefore attempted to realign them. To improve the chances of obtaining a successful alignment, we masked the A-to-I editing sites by an A→G transformation. Indeed, we discovered more than 700 ESTs ultra-edited in over 14,000 sites, which is about a third of the number of currently known editing sites. We deposited the coordinates of our sites in DARNED, the database of RNA editing. We also experimentally validated two of the targets.

As many apparent editing sites could really be sequencing errors, we applied stringent cutoffs and various cleaning procedures to ensure the sites we detected are genuine. The high confidence we have in our ultra-edited RNAs stems from the extremely small number of mismatch clusters of types other than A-to-G, because if our sites had resulted from a sequencing error, we would have observed a similar number of mismatch clusters of all types (or at least transitions). More evidence for the authenticity of the ultra-edited RNAs comes from their sequence motif, which is typical to editing by ADAR, and the localization of the editing sites in Alu elements. We believe that with relaxation of some of our strict detection thresholds, even more sites will be detected.

Characterization of the ultra-edited ESTs revealed that with the exception of a single liver library, the most edited tissue is the brain. However, this is to some extent because of the high coverage of the brain transcriptome; in terms of enrichment, the thymus, spleen, and muscle tissues are more ultra-edited, in agreement with previous observations [17]–[19]. Muscle tissue is ultra-edited in a couple of libraries despite the low expression of ADARs in that tissue [43], [64], [65]. Ultra-editing in muscle could thus be a result of induction of ADAR1, perhaps due to stress, as observed in [54]. The extreme number of ultra-edited RNAs from a regenerating liver library may also indicate induction of ADAR1 due to stress, possibly a viral infection [8]. However, the precise reason for ADAR's extreme hyperactivity in that sample remains to be elucidated.

The biological function of ultra-editing is still cryptic. Some of our findings (weak degree of sequence conservation, localization in new Alu subfamilies and in introns) may suggest that ultra-editing is generally undesirable, and that its major effect, if any, is gene-independent. In the latter case, the large amount of inosines in the transcriptome could affect gene expression globally, as recently shown [13], [33]. The other option is that ultra-editing affects the expression of specific genes. This could be mediated by modification of the RNA secondary structure (dsRNA destabilization), RNA nuclear retention, and cleavage/non-canonical splicing at the edited nucleotides. We demonstrated possible instances of the latter mechanism. The direct sequence changes induced by editing (A-to-G) do not seem to have an important function, in agreement with the large variation in the usage of editing sites that we experimentally observed (see Dataset S8). We did however find one ultra-edited RNA with five editing sites in a protein coding region (OLR1), four of which are non-synonymous. If more coding sequences are similarly ultra-edited, this could serve as an extremely powerful mechanism that (reversibly) diversifies protein sequences. Specific ultra-edited genes of interest are 17 genes involved in apoptosis and hematopoiesis, because of the role of ADAR1 in these processes [5], [52], [53], [56]. Regardless of the function of ultra-editing, the edited regions are characterized by potential to create particularly long, stable dsRNA structure, as expected from experiments with synthetic dsRNA [37], [38]. The stability of the dsRNA seems to be facilitated by a large frequency of repetitive elements, Alu and others, near the editing sites. It could however be that the editing efficiency is also affected by other factors, yet to be discovered.

Finally, our findings raise the intriguing question of how rare ultra-editing is. We detected a number of ultra-edited RNAs of the same order of magnitude as in previous genome-wide screens; as each ultra-edited RNA accommodates, by definition, a large number of sites, it could be that ultra-editing is responsible for a significant fraction of the cellular inosines. On the other hand, ultra-editing could be incidental, occurring sporadically in a stochastic manner. To decisively resolve this issue, editing must be studied in a transcriptome covered in depth. However, current technology and computational methods permit such studies only in small-scale [26], [66], [67]. We tend to adopt the view that ultra-editing is rare, for the following reasons. First, only 0.4% (3/695) of the ultra-edited regions are covered by four or more ESTs, compared to 10.6% (173/1637) in a previous genome-wide screen [17], [41]. Second, only 2/27 clones in our study, and 3/69 clones in [41], are far more edited than other clones. Third, Alu editing is, to a good approximation, a stochastic process where each site is edited independently with a given rate [41], [66]. Under this model, the probability to encounter an ultra-edited RNA is exponentially small. In the ultra-edited RNAs that we discovered, the editing rate was probably sufficiently large (due to e.g., particularly long dsRNA structure, specific induction of ADAR1, etc.) that ultra-editing was visible even with the current shallow coverage.

## Supporting Information

Dataset S1 A list of the accession numbers of the ultra-edited ESTs and their properties: coordinates of genomic origin; type, position, and count of mismatches; type and count of nucleotides neighboring the editing sites; and complete sequences of the aligning DNA and RNA. The list contains editing events of all types (A-to-G, G-to-A, A-to-C, etc.).
(TXT)

Dataset S2 A sorted list of the genomic coordinates of the ultra-editing sites (only A-to-G) formatted as a UCSC genome browser track (BED format).
(TXT)

Dataset S3 A list of accession numbers and basic properties (as in Dataset S1) of ESTs that are hyper-edited but not ultra-edited. These ESTs have passed all quality tests as the ultra-edited ESTs, but had less than 12 editing sites.
(TXT)

Dataset S4 A sorted list of the genomic coordinates of the hyper-editing sites (reported in Dataset S3), formatted as a UCSC genome browser track.
(TXT)

Dataset S5 A list of ultra-edited ESTs overlapping with exons, broken by 5′UTR, CDS, 3′UTR, and non-coding RNA.
(TXT)

Dataset S6 A list of functions enriched in ultra-edited genes. The file contains enriched GO and UniProt terms, gene counts, gene names, and P-values for enrichment as obtained from DAVID (http://david.abcc.ncifcrf.gov/). The file also lists the ultra-edited genes that are related to stress response, apoptosis, and hematopoiesis.
(XLS)

Dataset S7 A list of ESTs that overlap with both a UTR and an intron. The file contains the genomic coordinates and the names of the overlapping genes. Events of putative cleavage or non-canonical splicing are indicated, along with the accessions of mRNAs that support these events.
(XLS)

Dataset S8 A multiple alignment of the clones we experimentally sequenced for the two validated targets, with annotation and statistical analysis of the editing sites.
(XLS)

Figure S1 The chromatogram of the Sanger sequencing of the PCR product of DA098819 (A- green, C- blue, G- black, T- red). Editing sites are evident as nucleotides having an A in the reference genome and a G in the chromatogram (or signals for both A and G in the chromatogram). We annotated the editing sites with arrows. The level of editing (fraction of nucleotides with G at a given site) varies widely between the sites, indicating that the PCR product is heterogeneous, containing several differently edited molecules.
(TIF)

Figure S2 Same as Figure S1, for DA364252.
(TIF)

Table S1 A table describing the 12 sequence transformations used in the computational screen and the possible editing events detected by each transformation.
(DOC)

Text S1 A genome-wide analysis of the hyper-edited ESTs reported in Dataset S3.
(DOC)

Text S2 A note on experimental procedures, including all primers, kits, and experimental conditions, and the chromatograms of the sequenced PCR products.
(DOC)
