# Supplementary figures and images for: Identification of Widespread Ultra-Edited Human RNAs
Source: PLoS Genet. 2011 Oct 20;7(10):e1002317. doi: 10.1371/journal.pgen.1002317 (PMC3197674; doi:10.1371/journal.pgen.1002317)

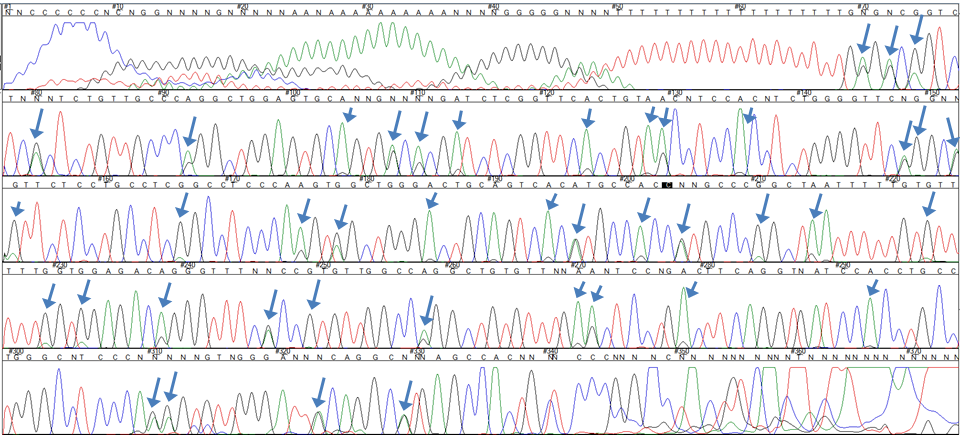

Supplement: Figure S1 — The chromatogram of the Sanger sequencing of the PCR product of DA098819 (A- green, C- blue, G- black, T- red). Editing sites are evident as nucleotides having an A in the reference genome and a G in the chromatogram (or signals for both A and G in the chromatogram). We annotated the editing sites with arrows. The level of editing (fraction of nucleotides with G at a given site) varies widely between the sites, indicating that the PCR product is heterogeneous, containing several differently edited molecules. (TIF) [file pgen.1002317.s009.tif]

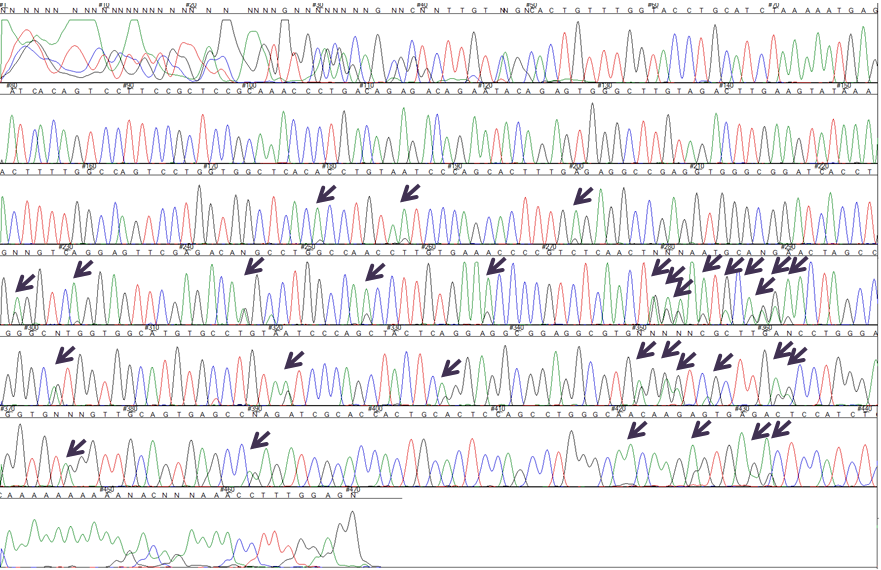

Supplement: Figure S2 — Same as Figure S1, for DA364252. (TIF) [file pgen.1002317.s010.tif]
