# Supplementary material for: Identification of Widespread Ultra-Edited Human RNAs
Source: PLoS Genet. 2011 Oct 20;7(10):e1002317. doi: 10.1371/journal.pgen.1002317 (PMC3197674; doi:10.1371/journal.pgen.1002317)
Supplement: Table S1 — A table describing the 12 sequence transformations used in the computational screen and the possible editing events detected by each transformation. (DOC) [file pgen.1002317.s011.doc]

Supplementary Table S1. **The 12 transformations applied to the DNA and candidate RNAs.**

| **DNA strand** | **RNA strand** | **Transformation** | **Detected editing events** |
| --- | --- | --- | --- |
| + | + | A→G | A-to-G, G-to-A, T-to-C, C-to-T |
| + | - | A→G | A-to-G, G-to-A, T-to-C, C-to-T |
| - | + | A→G | A-to-G, G-to-A, T-to-C, C-to-T |
| - | - | A→G | A-to-G, G-to-A, T-to-C, C-to-T |
| + | + | A→C | A-to-C, C-to-A, T-to-G, G-to-T |
| + | - | A→C | A-to-C, C-to-A, T-to-G, G-to-T |
| - | + | A→C | A-to-C, C-to-A, T-to-G, G-to-T |
| - | - | A→C | A-to-C, C-to-A, T-to-G, G-to-T |
| + | + | G→C | G-to-C, C-to-G |
| + | - | G→C | G-to-C, C-to-G |
| + | + | A→T | A-to-T, T-to-A |
| + | - | A→T | A-to-T, T-to-A |

**Legned:** The DNA strand is (+) if the transformation was applied to the (+) strand of the reference genome, and is (-) otherwise. The RNA strand is (+) if the transformation was applied to the sequence as it appears in GenBank (http://www.ncbi.nlm.nih.gov/genbank/) and is (-) if the sequence was reverse complemented prior to the transformation.
Editing events such as A-to-G and G-to-A, for example, can be distinguished based on the identities of the sequences bearing the As and the Gs (i.e., As in the DNA and Gs in the RNA: A-to-G; Gs in the DNA and As in the RNA: G-to-A). A-to-G and T-to-C cannot be distinguished based on the transformation and alignment algorithm: for example, an A-to-G mismatch in an alignment of DNA+/RNA+ can result either from A-to-G editing of the (+) strand or from T-to-C editing of the (-) strand.
