# Supplementary material for: Identification of Widespread Ultra-Edited Human RNAs
Source: PLoS Genet. 2011 Oct 20;7(10):e1002317. doi: 10.1371/journal.pgen.1002317 (PMC3197674; doi:10.1371/journal.pgen.1002317)
Supplement: Text S1 — A genome-wide analysis of the hyper-edited ESTs reported in Dataset S3. (DOC) [file pgen.1002317.s012.doc]

Supplementary Text S1: Properties of ESTs that are hyper-edited but not ultra-edited

In this document, we describe the results of rerunning our screen exactly as described in the Materials and Methods Section of the paper, but where the number of editing sites was allowed to be less than 12 (but at least five). We call the new ESTs detected this way “hyper”-edited, since they show a cluster of editing sites, but they are not ultra-edited.

After the filtering stage (see Figure 1A in the paper), we removed additional 77 ESTs that aligned to an rRNA gene on chr19. Several of the sites in this gene were genomic SNPs, and some ESTs better aligned to a region on chrUn_gl000220. We were therefore suspicious whether these ESTs represent a genuine editing event and decided to remove them.

After this additional filtering step, we remained with 280 hyper-edited ESTs. Below, we describe some of their properties.

**Table S2:** The number of hyper-edited ESTs of each mismatch type.

| A-to-G | 280 |
| --- | --- |
| G-to-A | 42 |
| A-to-C | 42 |
| C-to-A | 10 |
| G-to-C | 5 |
| A-to-T | 59 |
| Total number of non-A-to-G ESTs | 158 |

**Table S3:** The number of hyper-edited ESTs (A-to-G and G-to-A) with a given strand of the RNA sequence.

| A-to-G + | 164 |
| --- | --- |
| A-to-G - | 116 |
| G-to-A + | 37 |
| G-to-A - | 5^a^ |

^a^- P-value that this number is as low, based on the A-to-G mismatches, is 3×10^-5^.

**Table S4:** Statistics of the number of editing sites.

| Total number of sites | 2410 |
| --- | --- |
| Total number of unique sites | 2286 |
| Total number of novel sites | 2106 |
| Number of sites that are also ultra-edited | 103 |
| Number of sites overlapping with a genomic SNP | 14 |

**Table S5:** The percentage of each dinucleotide combination surrounding the hyper-editing sites.

| Upstream/Downstream | A | C | G | T |
| --- | --- | --- | --- | --- |
| A | 8.6% | 4.3% | 8.5% | 7.3% |
| C | 7.6% | 6.9% | 14.9% | 3.1% |
| G | 1.5% | 0.8% | 3.4% | 0.8% |
| T | 6.1% | 8.0% | 13.8% | 3.2% |

**Table S6:** The percentage of each nucleotide upstream and downstream of the hyper-editing sites.

|  | A | C | G | T |
| --- | --- | --- | --- | --- |
| Upstream | 29.0% | 32.8% | 6.6% | 31.1% |
| Downstream | 24.1% | 20.0% | 40.8% | 14.7% |

**Table S7:** The number of hyper-edited ESTs supporting each edited region.

| Number of different edited regions | Number of ESTs supporting each region |
| --- | --- |
| 251 | 1 |
| 12 | 2 |
| 1 | 5 |

**Table S8:** The number of hyper-edited ESTs overlapping with repeats.

| Number of ESTs overlapping with any repeat | 274 |
| --- | --- |
| Number of ESTs overlapping with an Alu element | 259 |

**Table S9:** The number of hyper-edited EST overlapping with genes.

| Number of ESTs overlapping with a gene | 196 |
| --- | --- |
| Number of ESTs overlapping with an exon | 19 |
| Number of ESTs overlapping with a 3’UTR | 14 |
| Number of ESTs overlapping with a 5’UTR | 1 |
| Number of ESTs overlapping with a coding sequence | 2 (RPS2, DEF6) |
| Number of ESTs overlapping with a non-coding RNA | 2 |

**Table S10:** The number of hyper-edited ESTs in each tissue (top tissues only). Enrichment was calculated as in Table 1 of the main text.

| Tissue | Number of ESTs | Enrichment |
| --- | --- | --- |
| liver | 62 | 6.993977177 |
| brain | 56 | 1.246682634 |
| prostate | 13 | 1.144909652 |
| uncharacterized tissue | 12 | 0.878076103 |
| muscle | 11 | 2.431786562 |
| eye | 11 | 1.322470445 |
| mixed | 10 | 0.723007752 |
| pancreas | 9 | 1.10423499 |
| lung | 9 | 0.661140798 |
| pineal gland | 7 | 23.11768238 |
| kidney | 7 | 0.829026302 |
| adipose tissue | 6 | 11.09727739 |
| connective tissue | 5 | 0.93164323 |
| bone marrow | 5 | 2.298049456 |
| uterus | 5 | 0.531122153 |
| thymus | 5 | 1.634071836 |
| intestine | 5 | 0.457805932 |
| spleen | 5 | 2.454403125 |
| ovary | 4 | 0.899472646 |
| mouth | 4 | 1.290134429 |
| lymph node | 3 | 0.817622295 |
| parathyroid | 3 | 3.859170148 |
| trachea | 3 | 1.607233319 |
| mammary gland | 3 | 0.377234973 |

**Table S11:** The number of hyper-edited ESTs in each health state (top health states only).

| Health state | Number of ESTs | Enrichment |
| --- | --- | --- |
| normal | 200 | 1.458541107 |
| mixed | 10 | 2.519472523 |
| head and neck tumor | 7 | 0.854891307 |
| non-neoplasia | 4 | 0.937714331 |
| lung tumor | 4 | 0.586700448 |
| colorectal tumor | 4 | 0.524203924 |
| prostate cancer | 4 | 0.510638189 |
| ovarian tumor | 4 | 1.136149255 |
| pancreatic tumor | 3 | 0.739954829 |
| breast (mammary gland) tumor | 3 | 0.551655226 |
| uterine tumor | 3 | 0.739258239 |
| germ cell tumor | 3 | 0.300425099 |
