# Supplementary material for: Identification of Widespread Ultra-Edited Human RNAs
Source: PLoS Genet. 2011 Oct 20;7(10):e1002317. doi: 10.1371/journal.pgen.1002317 (PMC3197674; doi:10.1371/journal.pgen.1002317)
Supplement: Text S2 — A note on experimental procedures, including all primers, kits, and experimental conditions, and the chromatograms of the sequenced PCR products. (DOC) [file pgen.1002317.s013.doc]

Supplementary Text S2: The experimental procedures

Amplification of the targets

We randomly selected two ultra-edited ESTs and designed primers to amplify genomic DNA and cDNA corresponding to these ESTs. The ESTs and their properties, the genomic coordinates, and the primers are given in the tables below.

| No. | EST | Num sites | Tissue | Library | Product coordinates | Strand | Product length | Known | Repeat | Gene |
| --- | --- | --- | --- | --- | --- | --- | --- | --- | --- | --- |
| 1 | DA098819 | 35 | Cerebellum | BRACE3 | chr19:53120654-53121052 | - | 399 | No | AluSx | ZNF83 |
| 2 | DA364252 | 25 | Subthalamic nucleus | BRSTN | chr2:242643526-242644012 | + | 487 | No | AluSq | ING5 |

| No. | EST | Forward primer (5’→3’) | Reverse primer (5’→3’) |
| --- | --- | --- | --- |
| 1 | DA098819 | CCCTGGACTGTGTAGCATGA | TTGAGGTGGTCAGAAATACAAAGA |
| 2 | DA364252 | CCAAGTTTCCCTCTCCCTTC | CCTCCAAAGGTTTTCTGTCTTT |

Genomic DNA and cerebellum RNA of a 66 years old male were purchased from a commercial vendor (BioChain Inc., Hayward, CA). cDNA was generated using Applied Biosystems High Capacity Reverse Transcription Kit, using random and oligo-dT primers, and PCR was performed on the genomic DNA and on the cDNA using KAPA Biosystems SybrFast at Tm=59⁰ for both targets.

Sequencing of the PCR products

We cleaned the PCR products using Takara Bio ExoSAP and submitted for Sanger sequencing. The genomic DNA was identical to the reference genome, except for a single SNP in the first target, which explains one editing site. The chromatograms of the cDNAs of DA098819 and DA364252 are given in Figure S1 and Figure S2, respectively. For both targets, we identified a large number of editing sites, indicated with arrows on the chromatograms. For DA098819, we identified 42 editing sites, validating all 34 sites present in the EST (not including the genomic SNP). For DA364252, we identified 32 sites, validating 18/25 of the sites of the EST.

Cloning of the PCR products

The editing sites seen in the sequences of the PCR products (Figures S1 and S2) have different editing efficiencies (fraction of RNAs edited at the site), indicating that not all RNAs were edited at all sites. We therefore cloned the PCR products into *E. coli* DH5α (Invitrogen pCRII-TOPO TA Cloning kit), and sequenced randomly selected colonies (plasmid isolated using Promega Wizard Plus SV or Invitrogen PureLink miniprep kits). We found the insert in 14 colonies in DA098819 and in 13 colonies for DA364252.

The multiple alignment of all clones is available as an Excel File with additional annotation in Dataset S8. For DA098819, we found 45 editing sites, 36 of which were supported by at least two cDNAs, and we validated 33/34 sites of the EST. The most edited clone had 36 sites. For DA364252, we found 38 editing sites, 30 of which in at least two cDNAs, validating 19/25 sites of the EST. The most edited clone had 22 sites. In Figure 6 of the main text, we plot the number of clones with each given number of editing sites. More statistics of the editing sites (including a `heatmap’ of the editing at each site/clone) can be found in Dataset S8.
